# Battle against novel coronavirus 2019-nCoV: International commitment to develop worldwide informing campaigns

**DOI:** 10.34172/hpp.2020.15

**Published:** 2020-02-16

**Authors:** Hamid Allahverdipour

**Affiliations:** ^1^Editor-in-Chief, Health Promotion Perspectives; ^2^Dean of Department of Health Education and Promotion, Tabriz University of Medical Sciences, Tabriz, Iran

On 13 February 2020, in Wuhan, China, 242 people died because of novel coronavirus 2019-nCoV.^1^ How long will it last and where else in the world, we don’t know. This newly emerged disease has made people wonder if and when they will be the next victims, and to what extent is the government responsible to protect them against. The World Health Organization (WHO) and China’s Ministry of Health have been actively fighting it. On 20 January 2020, the Director-General of the WHO, Dr. Tedros Adhanom Ghebreyesus, met President Xi Jinping of the People’s Republic of China to share the latest information on the outbreak and both reiterated their commitment to bring it under control and noted worldwide cooperation would be needed to deal with the disease effectively.^2^ It is estimated that 675 million US $ would be needed to cover the related expenses from February to April of 2020.^3^

Additionally, there have been several activities (e.g., media commitment to inform people, limited flights to and from China, checking travelers upon airport arrivals, and scientific efforts to produce a vaccine) to indicate a worldwide commitment. Our goal must be to learn from this experience and direct our efforts at controlling and preventing the emergence of a new viral disease in the future.

To develop a strategic plan to limit the human-to-human transmission of the virus, particularly in vulnerable countries most; to identify, isolate, and care for patients early; to communicate critical risk and event information; and to minimize social and economic impacts, the WHO has suggested the following actions^3^:

- Rapidly establishing international coordination and operational support.
- Scaling up country readiness and response operations.
- Accelerating priority research and innovation.

In addition to the WHO’s recommendations, because of various governments and their priorities, as well as differences in the economic infrastructures of developed and developing countries, more actions are needed. In fact, it is a moral responsibility of the developed world to assist the low-income countries regarding this important matter. Furthermore, the role of World Bank and international organizations can be instrumental in providing the needed resources to control the spread of the 2019-nCoV.

We suggest a World 2019-nCoV day or week to promote actions at the national level to inform the public. We hope politicians and governments, under the flag of the WHO, make an international commitment by the following slogan: “No one should die because of 2019-nCoV.”

## Ethical approval

Not applicable.

## Competing interests

The author declares that he has no competing interests.
